# Human wastewater tracking in tropical Hawaiian island streams using qualitative and quantitative assessments of combined fecal indicating bacteria and sucralose, an organic micropollutant of emerging concern

**DOI:** 10.1007/s10661-023-11545-7

**Published:** 2023-07-19

**Authors:** Carl J. Berg, John P. Alderete, Ethan A. Alderete

**Affiliations:** Kauai Chapter of Surfrider Foundation, P.O. Box 2195, Kapa’a, Hawaii 96746 USA

**Keywords:** Sucralose, Enterococci, Cesspools, FIB, Multiplication Rule, Tropical Islands

## Abstract

Prevalence of cesspools on tropical islands suggests that high concentrations of enteric bacteria in streams and coastal waters are an indicator of groundwater contamination by human wastewater. But enterococci bacteria may also be from homeothermic animals common to these watersheds or bacteria living in sediments. Sucralose, a manufactured chemical not destroyed in passage through the human gut, cesspools, septic systems, or wastewater treatment facilities, was used to test for the presence of human wastewater in streams on the island of Kauai, Hawaii. Effluent from six municipal wastewater treatment plants showed an average concentration of 39,167 ng/L of sucralose, roughly back-calculated to 9 ng/L per person, enough to present itself in cesspool effluent contaminated waters. Of 24 streams tested, 79% were positive for sucralose at least once in four sets of sampling. All streams tested positive for enterococci bacteria above established standards. Serial testing of the pair of indicators in the same location over time and applying the *Multiplication Rule* to the independent samples provide a probabilistic certainty level that the water is chronically polluted by human waste. When repeatedly paired with tests for enterococci, sucralose testing is a cost-effective means for assessing human health risk and for developing proper waste management programs that has been underutilized in under-developed tropical and island settings.

## Introduction

Tropical islands are classically defined by a small land mass surrounded by ocean with mountains catching rainfall and streams and rivers in well-defined and relatively short watersheds delivering the fresh water to coastal estuaries and beaches. Human settlements are typically established along the streams, using the fresh water for drinking, agriculture, fishing, and recreation. As the waterways pass through human settlements, the water can become polluted with agricultural runoff and human wastes from poorly designed or maintained onsite sewage disposal systems (OSDS) such as cesspools and septic systems. Polluted water then contaminates downstream sites including tidally influenced stream mouths, embayments, estuaries, coastal waters, and coral reefs. Communities living in island or rural mainland coastal areas are presented with public health risks from pathogens associated with both animal and human fecal matter.

Fecal indicating bacteria (FIB), e.g., *Enterococcus* spp*.* (ENT), *Escherichia coli* (*E. coli*), and total coliforms (TC), have been classically used as test organisms to identify public health risk from human pathogens associated with human wastes, with *Enterococcus* being the best surrogate for bacterial pathogens in tropical stream waters (Viau et al., 2011) and tropical marine recreational waters (Lamparellia et al., 2015), and have been adopted as criteria standards by states as recommended by federal government (U.S. E.P.A., 2018). Health risks include gastrointestinal illnesses as well as effects such as respiratory illness, skin rash, eye irritation, and ear infection (U.S. E.P.A., 2018)

In the tropics, routine monitoring of streams and coastal waters for FIB identifies areas that do not meet government standards but will not necessarily define the level of risk to the public from human-pathogen contaminated waters, as those bacteria may originate in fecal matter from homeothermic animals common to those watersheds, both feral and domesticated (Boehm et al., 2009; Fewtrell & Kay, 2015). Feces from cattle and dairy cows carry human pathogens and contaminate food crops through airborne transmission to soil and irrigation waters causing outbreaks of bacterial infections and product recall (Soller et al., 2010; Venegas-Vargas et al., 2016). Feral pigs are also known to carry human pathogens (e.g., Leptospires) that pass through feces and urine to surface waters (USEPA, 2009). Avian colonies are known to cause high concentrations of FIB (Vogel et al., 2013; Zimmer-Faust et al., 2020) mixed with human sources making quantitative microbial risk assessment problematic. Enterococci and *E. coli* bacteria are found in tropical soils and streams (Byappanahalli et al., 2012; Ekklesia et al., 2015; Goto & Yan, 2011; Hardina & Fujioka, 1991; Luther & Fujioka, 2004; Viau et al., 2011) and are capable of colonizing and growing in Hawaii’s soils (Byappanahalli et al., 2012).

Because human and non-human sources of FIB pose varying degrees of risk to public health and require different management strategies to alleviate that risk, it is important to determine if human wastewater is present in recreational waters. In fact, some regulations require different public notification procedures to be followed depending on whether human sources of FIB are determined (Hawaii Department of Health, 2021). But pathogens themselves are both difficult and dangerous to culture and quantify. Organic micropollutants (OMP) including pharmaceuticals, personal care products, and artificial sweeteners from anthropogenic sources (e.g., on site disposal systems [OSDS] [cesspools, septic systems], or wastewater treatment plants [WWTP]) are frequently used as tracers for detecting and identifying sources of wastewater. A recent review of source tracking in tropical Hawaii using these methods is found in Johnson (2020).

Beginning in 2014 studies began combining microbial (FIB) and chemical fecal indicators (artificial sweeteners) in analysis of recreational waters (Ekklesia et al., 2015; Guérineau et al., 2014; Sima et al., 2014). Ideally the OMP indicator should be (1) source specific for raw wastewater or treated effluents, (2) ubiquitous (> 80% detection frequency) in contaminated waters of concern yet missing in background samples, (3) concentrated in samples with respect to levels of detection, (4) persistent in subsurface groundwaters and (5) detected with rapid, inexpensive yet sensitive analysis (McCance et al., 2018; Oppenheimer et al., 2011; Soh et al., 2011; Spoelstra et al., 2013; Yang et al., 2017).

Sucralose (SUC) is one such OMP that has stood out, as it is ubiquitous in diets worldwide, present now in relatively high concentrations in wastewater, and easy to analyze. The use of SUC in human food products and beverages is historically well documented (Brorström-Lundén et al., 2008; Molinary & Quinlan, 2006). SUC is a man-made chemical (C_12_H_19_Cl_3_O_8_) introduced as an artificial sweetener approved by the US Food and Drug Administration in 1998, used in Europe by 2003 (Loos et al., 2009; Robertson et al., 2016) and widely adopted worldwide thereafter as a substitute for sugar in food manufacture. Its use is increasing worldwide (Alves et al., 2021). SUC was shown to pass through the human body unchanged; 85.5% with feces and 11.2% with urine over 5 days (Roberts et al., 2000) making it an ideal indicator of human waste associated pathogens. Soon after introduction with food, its presence in wastewater, resistance to treatment in WWTP, and general suitability as a qualitative and quantitative tracer of human wastewater was examined and reviewed repeatedly (Bernot et al., 2016; Biel-Maeso et al., 2019; Mawhinney et al., 2011; Oppenheimer et al., 2011; Van Stempvoort et al., 2020; Yang et al., 2018).

SUC is hydrophilic (Bernot et al., 2016) refractory (Yang et al., 2018), and recalcitrant with less than 15% removal by adsorption, biodegradation (Badruzzaman et al., 2013), or photolysis (Sang et al., 2014; Tran et al., 2014). There are many studies that address its properties, including its low adsorption (Biel-Maeso et al., 2019), persistence in soils (Biel-Maeso et al., 2019; Van Stempvoort et al., 2020), low biodegradation in the environment (Labare & Alexander, 1993; Tollefsen et al., 2012), and source specificity (Oppenheimer et al., 2011; Yang et al., 2018).

Additionally, the utility of SUC as a human waste tracer is supported by straight-forward analysis and low minimum detection limits (MDL) using previously established combinations of solid-phase extraction (SPE) and liquid chromatography-tandem mass spectrometry (LC-MS-MS). Methods used for determining concentrations in water and wastewater matrices were first reported in early 2008 in Swedish studies (Brorström-Lundén et al., 2008) and subsequent studies (Arbelaez et al., 2015; Batchu et al., 2013; Batchu et al., 2015; Loos et al., 2009; Loos et al., 2013; Minten et al., 2011; Morlock et al., 2011; Ordóñez et al., 2012; Scheurer et al., 2009). There are reviews of LC-MS-MS methods specifically for sweeteners (Lange et al., 2012; Lorenzo Ferreira et al., 2018; Luo et al., 2019). New methods have been developed for SPE (Lakade et al., 2018) and for on-line high performance SPE-LC-Ms-MS (Henderson et al., 2020).

Although the use of SUC as a tracer of surface and groundwater contamination by human wastewater is well established in developed nations over the 20 years that SUC has been used as a food supplement and the concentrations have increased with more widespread use, no published reports were found of SUC concentrations for WWTP in small tropical islands. Studies have been done using SUC for identification of contamination by OSDS of groundwater (Edwards et al., 2019) because of concern for contamination of underlying drinking water aquifers and of surface water (Edwards et al., 2017) on the Caribbean Island of Barbados and post-hurricane drinking waters of Puerto Rico (Bradley et al., 2021; Lin et al., 2020). A study of coastal nutrient enrichment in Vatia Bay, Samoa, using the combination of caffeine and SUC, showed those tracers of human wastewater were present in both the stream and bay (Whitall et al., 2019). A US Geological Survey (USGS) review of wastewater source tracking in Hawaii highlighted the use of pharmaceutical and organic waste compounds in 21 studies over the four main islands (Johnson, 2020), with caffeine most frequently used (Knee et al., 2010; McKenzie et al., 2021) and artificial sweeteners inexplicably neglected. This study on the small (1456 sq km) tropical island of Kauai followed the recommendation of combining FIB and wastewater OMP indicators in the tropics (Ekklesia et al., 2015; Guérineau et al., 2014). Concentrations of SUC were measured in municipal WWTPs and in surface waters from streams around the island, in combination with FIB, as an easy, relatively inexpensive, well-established method to determine which streams were polluted with human wastes and required further investigation as presenting a public health risk.

## Materials and methods

### Sampling SUC in municipal wastewater

Six municipal wastewater treatment plants (WWTP) on Kauai were sampled between 8/26/2020 and 8/27/2020 and one on 12/01/2020 (Table 1). Latitude and Longitude of site locations were derived from Google Earth Pro Images © Maxar Technologies.

**Table 1:** Date, time, and location of sampling from WWTP in six communities on island of Kauai and one on Hawaii

	Date	Time	^o^ North latitude	^o^ East longitude
WWTP Sites, Kauai Is.				
Wailua WWTP	8/26/2020	12:00	22.039421	−159.3368
Poipu WWTP	8/26/2020	13:20	21.880193	−159.4628
Princeville WWTP	8/27/2020	08:12	22.217653	−159.4925
Waimea WWTP	8/26/2020	14:00	21.96384	−159.6783
Lihue WWTP	8/26/2020	11:00	21.968608	−159.3498
Puhi WWTP	12/1/2020	10:05	21.957692	−159.3719
Kealakehe, Hawaii Is.	9/16/2019	10:00	19.660529	−156.0161

Grab samples were taken by a WWTP employee from final discharge tanks of R-1 treated water before it left the facility. Effluent R-1 water has been oxidized, filtered, and disinfected to meet highest standards of water quality in reduction of bacterial and viral pathogens to permit recycling (Hawaii Department of Health, 2016). All were disinfected by UV light. Grab sample water was poured into 1-liter certified clean amber glass wide mouth jars, sealed, immediately submerged in ice, and taken to the laboratory facility. There jars were repeatedly inverted for mixing before pouring approximately 75 mL into two IDEXX polystyrene 120 mL jars and frozen in a −20^o^ residential type freezer. These paired samples were kept frozen and shipped in insulated containers, under dry ice, to the analytical laboratory at Florida International University. Data from a WWTP in Kealakehe, Hawaii island, for the community in Kona was from Bennett (2020). Samples were collected in the same manner and analyzed by the same laboratory as Kauai samples.

Each WWTP provided discharge rate on the day of sampling and the average discharge rate for the entire month of the day of sampling. Sampling was conducted during period of COVID-19 pandemic when there was almost no tourism, hotels were closed, and sewage was mainly collected from residential areas. WWTP provided the number of housing units serviced, and the County of Kauai general plan (SMS, 2014) provided average number of persons occupying each house for each community. An approximate number of individuals contributing to WWTP effluent were derived from this information.

### Sampling SUC in stream waters

Twenty-four perennial stream sites were each sampled four times from 6/7/2020 to 4/25/2021 (Appendix Table 7). These were chosen as they ranged along 2/3rds of the Kauai coastline and were accessible by automobile. They were not chosen by any previous knowledge of bacterial contamination. Each site was sampled twice during the dry season (May 1 through October 31) and twice during the wet season (November 1 through April 30), as defined in Hawaii Department of Health (2021). Sampling was done by two teams on mornings of low tides. One team sampled north and east shorelines: the other south-east to west coastlines. Sampling was done near the stream mouth. Samples were collected by attaching two IDEXX polystyrene 120 mL jars to the end of a pole and submerging them to approximately 15 cm until they were full. When removed from the water they were capped, labeled, secured with a custody seal and put on ice. Samples were shared among the two teams, so that each team got one sample for bacterial analysis at each team’s laboratory.

### Laboratory analysis for SUC

Samples were analyzed for SUC by online solid phase extraction coupled to high-resolution mass spectrometry at Florida International University, Environmental Analysis Research Laboratory, using techniques developed at that laboratory (SOP-2014-0-130.1, Batchu et al., 2015). The method detection limit (MDL) establish by the laboratory for stream samples was 12.1 ng/L.

### Laboratory analysis for fecal indicating bacteria

Water samples were collected and analyzed as described in a Quality Assurance Project Plan submitted to Hawaii Department of Health, specifically for analysis of salinity and enterococci concentrations. Enterococci were measured using IDEXX Enterolert™ defined substrate technology with positive and negative controls (EPA SM9230D). *E. coli* and total coliforms were detected using same methods but using Collilert™ as described by IDEXX. Values were conventionally reported as Most Probable Number per 100 ml of sample water (mpn/100 ml) which is interchangeable with colony-forming units (cfu).

### Laboratory analysis for salinity

Salinity of each stream sample was measured in parts per thousand, by inserting a distilled water rinsed EXTECH AZ 8371 electronic water quality meter probe into each sample bottle after a 10 mL sample was removed for analysis for enterococci.

### Statistical methods

Microsoft Excel was used for all statistical analyses. Data were divided into five groups set by the number of times sucralose was detected during four repeated samplings (i.e., *groups 0, 1, 2, 3, 4*). This was done to determine the minimal number of independent samples required to be confident that simple presence of sucralose was a reliable indicator of human fecal contamination of stream water. When sucralose concentrations were reported as below the 12 ng/L limit of detection, then a value of 6 ng/L (halfway between zero and 12 ng/L) was used so that a geometric mean could be calculated for all groups. When a bacteria concentration exceeded the 24,196 mpn/100 mL upper limit of detection, then the absolute value of 24,196 mpn/100 mL was used. Median descriptive statistics were used due to small group sample size, but geometric means were also calculated as is standard for analysis of bacteria concentrations with large ranges of values.

Comparisons among groups used Excel’s single factor ANOVA for multiple samples with a *p*=0.05. Bonferroni’s multiple comparison test for five multiple samples dictated *p*=0.01 be used as test for significance. Post hoc analysis of differences among groups for test parameters (sucralose, bacteria, and salinity) was done using Excel’s Student *t*-test with 2-tails and assuming unequal variance.

Probability analysis used the *Multiplication Rule P*(*A and B*) = *P*(*A*) ∗ *P*(*B*) for independent events where two events A and B are independent if the fact that A occurring does not affect the probability of B occurring. In this study stream, sample A is independent of sample B from the same stream because concentrations of sucralose and enterococci bacteria are dependent on human fecal contamination on widely different occasions and different stream flow. The fact that sucralose presence in sample A was not necessarily followed by sucralose in sample B supports this.

The *Multiplication Rule* applies for situations where A = presences of sucralose >LOD and B = presences of *Enterococcus* >statistical threshold value of 130 cfu per 100 ml of sample. They are independent because (1) the events actually co-occur and (2) the occurrence of one event does not influence the occurrence of the other. The human ingestion of SUC is independent of the presence of fecal indicating bacteria in the human feces that are contributing to stream pollution. Fecal contamination may come from feral animals that never ingest SUC.

## Results and discussion

### SUC in municipal wastewater

SUC concentrations in WWTP discharge water ranged from 22,853 to 62,352 ng/L with a mean value of 39,167 ng/L (Table 2) which defines the community level of consumption and discharge of SUC by individuals on Kauai. The 42,323 ng/L in the WWTP discharge from Kona community, on Hawaii island, is similar (Bennett, 2020).

**Table 2:** SUC concentrations, volume of discharge, and grams of SUC discharged from six community WWTP on Kauai and one from Kona, Hawaii Is. for comparison. Listed in order of increasing concentrations

Location	Sucralose concentrations (ng/L)	WWTP discharge (L/day)	Sucralose discharge (grams/day)
Kauai Island			
Wailua	22,853	1,105,340	25.3
Poipu	26,638	840,403	22.4
Princeville	31,167	949,457	29.6
Puhi	39,326	1,522,599	59.9
Waimea	52,667	713,520	37.6
Lihue	62,352	3,793,361	236.5
Mean value	39,167	1,487,447	58.3
Hawaii Island			
Kona	42,333	6,813,741	288.4

Among four mainly single-family residential housing communities (Princeville, Puhi, Lihue, Poipu), a mean concentration of 9 ng/L per person was calculated from the number of housing units being serviced by the WWTP multiplied by the estimated average number of people occupying each unit (SMS, 2014) (Table 3). Concentrations are higher for Wailua and Waimea because of inaccurate estimates of occupancy of commercial units and multifamily dwellings.

**Table 3:** Calculated concentration of SUC per person in municipal WWTP effluent for each community, listed in increasing concentrations

Community	(ng/L)/person
Princeville	8.35
Puhi	8.74
Lihue	9.47
Poipu	9.55
Wailua	29.9
Waimea	43.7

### SUC in stream waters

The results of stream sampling were placed into groups by the number of times SUC was detected over four samplings (Appendix Table 7). Of a total of 96 stream water samples, SUC concentrations ranged from less than the method detection limit (12.1 ng/L to 2157.5 ng/L). The median and geometric mean values for the entire sampling were derived using a value of 6 ng/L where values were <MDL (Table 4).

**Table 4:** Results of the total sample collection for SUC, bacteria and salinity. Count indicates the number of samples analyzed for that parameter. Geomean = geometric mean

	Sucralose	Enterococcus	*E. coli*	T. coliforms	Salinity
	ng/L	mpn / 100 ml	mpn / 100 ml	mpn / 100 ml	ppt
Count	96	88	86	86	96
Minimum	<12	30	85	5,172	0
Maximum	2157	12,997	>24,196	>24,196	31.7
Median	6	463	321	24,196	0.6
Geomean	20	478	421	20,465	0.6

Data was sorted into five groups based on the number of times SUC was detected over four samplings for each stream site (Table 5)*. Groups 0, 1, 2, 3,* and *4* comprise 20.8%, 20.8%, 25.0%, 12.5%, and 20.8% of the 24 sites, respectively.

**Table 5:** Median and Geometric mean values for each group based on the number of times the sites tested positive for SUC. Entero = *Enterococcus* bacteria

# Times sucralose positive	# Sites in group	Sucralose ng/L	Entero mpn/100 ml	*E. coli* mpn/100ml	Total coliforms mpn/100ml	Salinity ppt
Median						
0	5	6	353	206	24,196	0.29
1	5	6	350	299	>24,196	0.32
2	6	9	375	232	24,196	1.54
3	3	24	428	279	24,196	0.3
4	5	201	1050	842	>24,196	6.3
Geometric mean						
0	5	6	299	222	19,119	0.29
1	5	9	540	486	22,801	0.39
2	6	16	375	344	19,940	0.86
3	3	19	392	293	17,230	0.43
4	5	224	1132	1201	22,730	1.87

Comparisons among groups using single factor ANOVA for multiple samples with Bonferroni’s multiple comparison test for five multiple samples set *p*=0.01 for differences that were significant. There were significant differences for SUC (*p*= 1.74E-07) and salinity (*p*= 1.40E-05) within the groupings, whereas there was no significance (*p*= 0.13, 0.04, 0.07) for the fecal indicating bacteria enterococci (ENT), total coliforms (TC) and *E. coli*, respectively.

Post hoc analysis of differences among groupings for test parameters (SUC, bacteria and salinity) was done using Excel’s Student’s *t*-test with 2-tails and assuming unequal variance. With the Bonferroni correction, now *p*=0.01, SUC differences for *group 4* vs g*roups 0, 1, 2, and 3* were significant. Differences for *group 3* vs *groups 0, 1, and 4* were significant. No other comparisons were significant.

### Bacteria in stream waters

#### Enterococci

ENT concentrations compiled from all stream samplings (Appendix Table 7) indicate that overall, the streams were polluted with ENT and *E. coli* bacteria at concentrations that exceed USEPA and State of Hawaii standards for recreational waters (Table 6), but there are no standards for TC as they are ubiquitous in tropical aquatic ecosystems.

**Table 6:** USEPA recommended criteria for fecal indicating bacteria in marine and fresh water, where GM = geometric mean, STV = statistical threshold value. Adapted from: https://www.epa.gov/sites/production/files/2015-10/documents/rec-factsheet-2012.pdf

Criteria elements		Estimated illness rate 36/1000	
Indicator	Waters	GM (cfu/100 ml)	STV (cfu/100 ml)
Enterococci	Marine & fresh	35	130
*E. coli*	Fresh	126	410

The geometric mean (GM) of ENT for the combined samples was 478 mpn/100 ml (Table 4), greater than the 35 mpn/100 ml EPA standard. The statistical threshold value (STV) of 130 mpn/100, which shall not be exceeded by more than 10% of samples, was exceeded in 90.9% of the composite samples. As a group, these streams would be classified as polluted with ENT.

Looking at each stream separately, geometric means for each were >35 mpn/100 ml by 3.8 to 224 times, and all exceeded the 130 mpn/100 STV >10% of the time, ranging from 50 to 100% of the time, although these values are based on only 3–4 samples. Thus, each stream would be considered as polluted with ENT. The concentrations of ENT bacteria among all groups were not different from one another with the Bonferroni adjusted *p*=0.01.

##### *Escherichia coli*

*E. coli* standards for fresh waters are a geometric mean of 126 mpn/100 ml and a STV of 410 mpn/100ml (Table 6). The geometric mean for combined samples was 421 mpn/100 ml (Table 4) and values exceeded the STV 39.5% of the time. Looking at each stream separately, geometric means for each were >126 mpn/100 ml, except for Limahuli stream (104 mpn/100 ml). Three streams in each of *group 0, 1, and 2* and one in *group 3* had no *E. coli* > STV of 410 mpn/100 ml.

Therefore, in general, most streams would be considered polluted for E. coli except those two streams in *group 0* with low GM values (Limahuli=104 mpn/100 ml, Lumahai=130 mpn/100ml) and no single values >STV=410 mpn/100ml. The concentration of E. coli bacteria among all groups were not different from one another with *p*>0.04 against the Bonferroni adjusted *p*=0.01.

### Total coliforms

There is no EPA standard for TC as they are ubiquitous in natural waters. TC geometric mean is 20,465 mpn/ 100 ml for 86 tests with all streams having at least one sample >19,863 mpn/100 ml. Of total samples, 70.9% were at or above 24,196 mpn/100 ml, the limit of detection. There were no differences in group values as *p*>0.11 for all inter-group comparisons.

### Salinity of stream waters

Because most samples were taken near stream mouths and at low tides, salinity of the water varied with respect to stream flow, groundwater intrusion, and saltwater inundation by waves. Three streams (Lumahai, Wainiha, Wailua) were sampled >100 m upstream and would be considered freshwater by Hawaii standards (Hawaii Dept. Health, 2021), as ion concentration was always below 0.5 ppt. Limahuli and Waikomo streams were sampled above coastal waterfalls, so no saltwater intrusion or mixing was possible. All others would be considered as brackish water. Sample salinity varied from 0.0 to 31.7 ppt with a GM concentration of 0.59 ppt for 96 tests (Table 4). Salinity in *group 4* samples were different than that for *groups 0, 1, and 3* at *p*< 0.003 but only at *p*=0.03 for *group 2*.

## Discussion

It appears that SUC has not been used as a tracer of human wastewater contamination in tropical under-developed areas, especially islands, because of the assumption that local diets would not be consuming quantities appreciable enough to be detected in recreational waters (e.g., Edwards et al., 2017). Analysis of samples from six WWTP on Kauai showed that SUC was present in community wastewater treatment plant effluent at a mean value of 39,167 ng/L, setting an approximate value for raw sewage in OSDS and a calculated value of 9 ng/L/person served, considering that not every person in the area consumes and passes SUC with their feces directly to the WWTP. The conservative use of 9 ng/L/person allows prediction of average levels of SUC concentration to be expected in streams with known numbers of human occupants in the watershed for quantitative assessment of levels of contamination. SUC concentrations in Kauai streams ranged from less than the method detection limit (12.1 ng/L) to 2157.5 ng/L, with a geometric mean of 20.2 ng/L. Samoa streams ranged from 12.79 to 369.8 ng/L with a geomean of 55.7 ng/L (Whitall et al., 2019). Puerto Rico tap drinking water ranged from 2.9 to 859.4 ng/L with a median value of 18.3 ng/L (Lin et al., 2020), while Bradley et al. (2021) reported sucralose ranging from non-detect up to 2100 ng/L (median was non-detect) in 7 commercial locations, but no detections in residential tap samples. Barbados surface waters had positive mean values of 3 to 19 ng/L (Edwards et al., 2017). This study and the review of concentrations of SUC in tropical and sub-tropical areas in recent times establish SUC utility as a quantitative measure of human wastewater contamination of drinking and recreational waters.

SUC testing also has merit as a simple and inexpensive *qualitative* measure of human wastewater contamination. When SUC results are considered simply as binary readings, the probability of just the presence/absence occurring can be calculated by the *Multiplication Rule* of probability where *P*(*A and B*) = *P*(*A*) ∗ *P*(*B*) for independent events, in this case repeated sampling of a site.

With each sampling event, the probability of getting a positive measure of SUC >MDL by chance alone is *p* = 0.50 and for four independent samples all being positive *p* = 0.5 ∗ 0.5 ∗ 0.5 ∗ 0.5 = 0.0625; a comfortable level of certainty of contamination with a minimal level of sampling.

ENT concentrations >MDL results can also be considered as binary samples. If four samples are positive for the presence of ENT, then again *p* = 0.0625 that the FIB are present not by chance. When the mere presence or absence of a STV of 130 mpn/100 ml is used as the threshold for a positive binary response, then there is rational for declaring the stream a public health risk based on EPA standards.

The *Multiplication Rule* of probability can be used to calculate the probability of both SUC and ENT being present in samples by chance alone, since the measures are independent from one another. SUC concentrations for the entire sample (*n* = 88) were poorly correlated with paired ENT concentrations (*r* = 0.015). SUC concentrations are independent of fecal indicating bacteria (FIB) concentrations, as levels of SUC ingestion occurs without respect to gut bacterial loads. SUC can occur in waters without FIB detected, e.g., where bacteria have been killed by an effective wastewater treatment system (UV light or chlorination) or have been removed by mechanical filtration or the ground.

FIB concentrations are not dependent on SUC being present. There can be very high levels of ENT present in streams, but no detectable SUC, as none was present in the watershed. High ENT concentrations may be solely due to animal fecal matter, because human sources of FIB were not ingesting SUC, because the initial SUC load was low and highly diluted by stream volume while fecal bacteria load was high or because bacteria were established and growing in the stream bed.

For single sample positive binary presence of both independent indicators SUC *p* = 0.5 and ENT *p* = 0.5 therefore *p* = 0.5 ∗ 0.5 or *p* = 0.25 that this combination was not occurring by chance. With only two sets of samples, a probability of *p* =0.0625 is obtained, warranting more testing and source tracking. If all four samples are >MDL for both indicators, then *p* = 0.25 ∗ 0.25 ∗ 0.25 ∗ 0.25, *p* =0.0039 gives a high level of confidence that the stream was polluted with human fecal indicators, pathogens are highly likely present, and contamination should be acknowledged as such for management and legal purposes.

Five examples from *group 0,* where SUC was never found, are two streams with no human habitation (Lumahai and Limahuli) so that only animal fecal matter would be present; one which has two families on septic system away from stream and they may not consume any SUC (Waikoko) and two (Hanapepe and Waimea rivers) which have high volumes of discharge and adjacent homes are mostly serviced by County WWTP that discharge into deep injection wells.

In *group 1*, where SUC was found only once per stream, the four streams (Hanamaulu, Kalihiwai, Waioli, Waiopili) drain rural areas that have few inhabitants living by the stream, who may not have been in residence at periods of sampling (Appendix Table 7).

Rural communities near larger rivers in *groups 1, 2, and 3* (Hanalei, Huleia, Wailua) were not serviced by WWTP, and all rivers had low concentrations of SUC (medians of 31 ng/L, 31 ng/L, and 35 ng/L, respectively) and ENT concentrations (medians of 303 mpn/100 mL, 322 mpn/100 mL, 222 mpn/100 mL respectively) (Appendix Table 7), perhaps reflective of large discharge volume of the rivers compared to the streams. These findings may also be indicative of effective treatment for ENT by OSDS treatment which kills most bacteria but does not degrade SUC. Most cesspools along the Hanalei River had been replaced with septic systems in a dedicated and effective effort to reduce ENT concentrations.

**Fig. 1:**
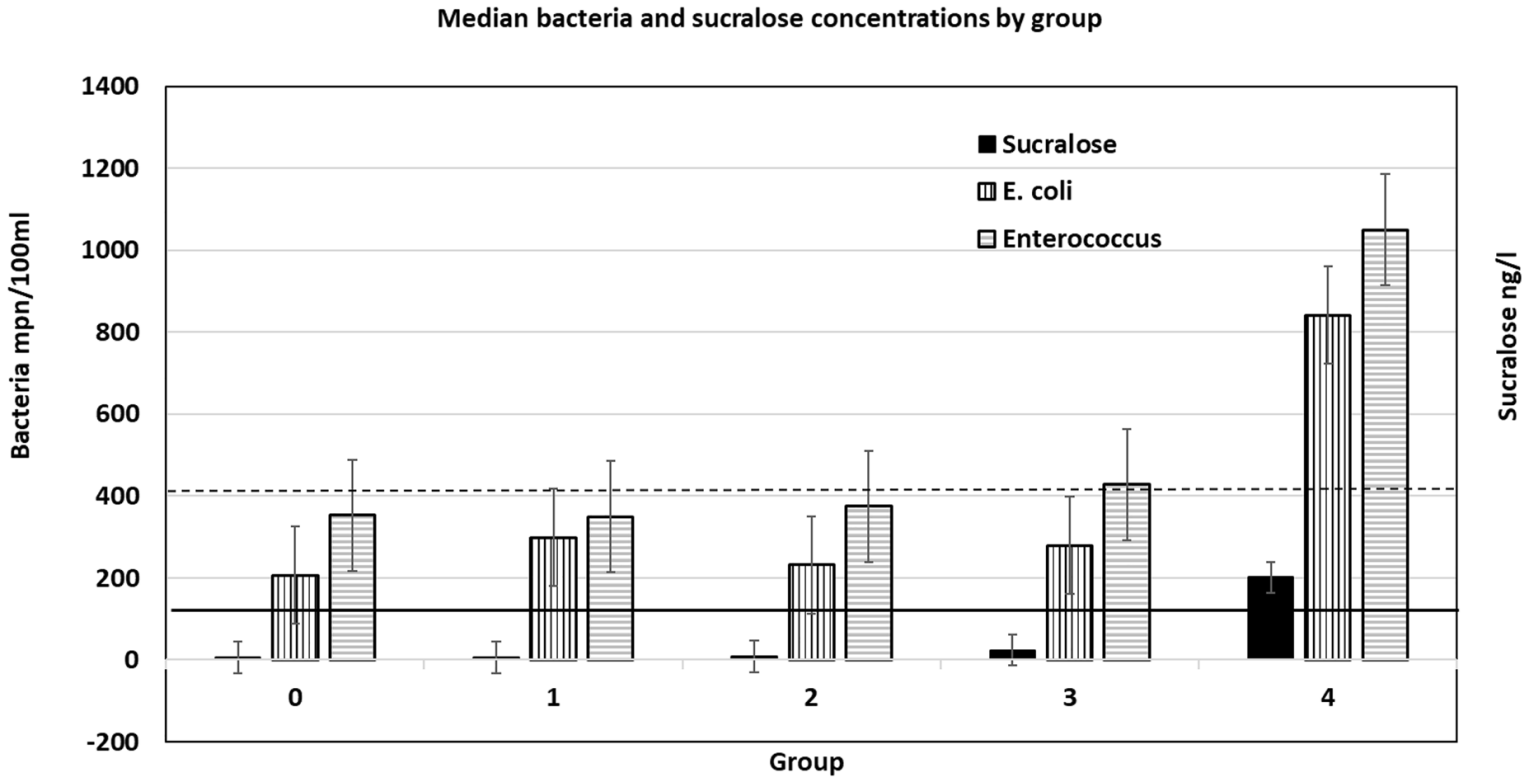
Median concentration values of bacteria and SUC plotted by number of times SUC was detected in each group. Numerical values are the same for both *Y*-axis. Error bars are present for each group. Solid horizontal line is the EPA STV for ENT (130 cfu/100 ml). Dashed horizontal line is the EPA STV for *E. coli* (410 cfu/100ml).

Generally, although SUC concentrations in *groups 1, 2, and 3* were low (Table 5), they indicate human sewage was present and correspond to an overall geometric mean value for ENT 3.3 times above the STV (Fig. 1). Where SUC concentrations were greater than 200 ng/L, there was a sharp increase in bacteria concentrations (Fig. 1). Streams in *groups 0, 1, 2, 3, and 4* were above the ENT STV 85%, 89%, 91%, 93%, and 100% of the times, respectively. Thus, all were polluted with fecal indicating ENT bacteria, but the lack of SUC detections indicated no, or limited, impacts from human sewage in group 0 streams.

Geometric means of *E. coli* concentrations were only 0.9 of its STV (Fig. 1). Whereas the median values for EC were below the STV of 410 mpn/100 ml for all but *group 4*, the STV was exceeded by 10.5%, 33.3%, 28.6%, 36.4%, and 94.1% of the streams *in groups 0, 1, 2, 3,* and *4*, respectively. Thus, EC is an equivocal indicator of fecal bacteria. Because there is no EPA standard for TC and they were ubiquitous and present in all samples at concentrations >5218 mpn/100mL (Table 4), they too are not a useful indicator of fecal contamination in recreational waters.

## Conclusions

Linking the organic micropollutant tracer SUC, with the fecal indicator bacteria ENT, provides a simple and accurate indication of the risk of human pathogens in drinking and recreational waters. Serial testing of the pair of indicators in the same location over time and applying the *Multiplication Rule* to the independent samples will provide a probabilistic certainty level that the water is chronically polluted by human waste, thus requiring management action. With a minimum of four repeated samplings at a site, the pairing becomes a strong and easily used method for qualitative public risk assessment. Continued development of cost-effective, rapid, and deployable technologies for SUC and/or ENT is needed for timely water quality testing and decision-making. When repeatedly paired with tests for ENT, SUC is a cost-effective means for assessing risk of human wastewater contamination of recreational waters that has been underutilized in under-developed tropical island settings.

## Figures and Tables

**Table 7:**
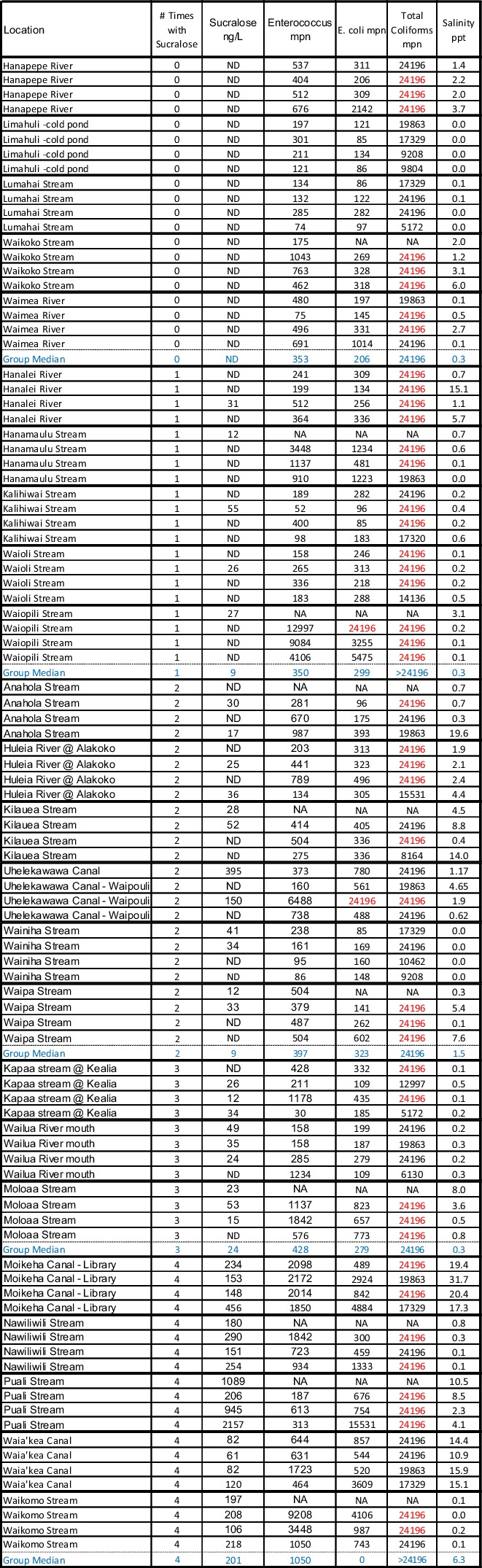
Results of stream sample analysis grouped by the number of times SUC were detected in four samplings. Numbers in red represent bacteria concentrations greater than the limit detection (>24,196 mpn) that were transformed to a value of 24,196 for statistical purposes. Numbers in blue indicate median values for that group

## Data Availability

All data is provided in Appendix Table 7. Additional information is freely available from corresponding author.
